# The usage of DiGA, stratified by sociodemographic/socioeconomic characteristics

**DOI:** 10.1093/eurpub/ckac131.180

**Published:** 2022-10-25

**Authors:** N Hannemann, B Babitsch

**Affiliations:** New Public Health, Osnabrück University, Osnabrück, Germany

## Abstract

**Background:**

The German government undertakes efforts to implement DiGA into the statutory health insurance to improve its quality. DiGA are physician-prescribed applications for patients with certain diagnosed diseases, whose costs are covered by the statutory health insurers. DiGA have the potential to improve healthcare, but it is also possible, that the usage of these applications perpetuates existing health inequalities, summarized by the term Digital Divide; meaning that socially deprived populations are less able to benefit from digital technologies. The aim of this analysis is to determine whether differences exist in DiGA use by sociodemographic/socioeconomic characteristics.

**Methods:**

The results based upon the analysis of an online survey involving 1,200 people (18-74 years) living in Germany between March 10 and March 18, 2022. The sample composition reflects the current distribution of age, gender, and place of residence in the federal states (uncrossed). The questionnaire focused, among other aspects, on the use of DiGA. A binary logistic regression was used for the analysis.

**Results:**

Compared to the lowest subjective social status (SSS), probands with a medium (OR 2.865) or a high SSS (OR 4.085) are more likely to use DiGA. Compared to the reference group (60 years and older), the 18-29-year- (OR 2.044) and the 30-39-year-olds (OR 1.952) tend to have a higher likelihood of using DiGA. The likelihood of the use decreases among probands with medium (OR 0.632) and high educational degree (OR 0.580) compared to the reference group (low education).

**Conclusions:**

In accordance with the results of existing studies, social differences could be identified regarding known determinants of health inequalities, like age and SSS. In this analysis, the highest degree of education does not appear as a predictor for an increased likelihood of use. Thus, further analyses are needed to address the influence of education, especially to develop a broader understanding of the DiGA use.

**Key messages:**

• It appears that DiGA are not equally accessible or used across different population groups, and thus indicating an already existing or emerging Digital Divide regarding the use of DiGA.

• Contrary to the broad assumption that higher expressions of health determinants are related to a higher likelihood of using DiGA, a higher degree of education decreases the likelihood of using DiGA.

